# Heritable genetic variation but no local adaptation in a pine-ectomycorrhizal interaction

**DOI:** 10.1007/s00572-020-00941-3

**Published:** 2020-02-20

**Authors:** Jim Downie, Jonathan Silvertown, Stephen Cavers, Richard Ennos

**Affiliations:** 1 Institute of Evolutionary Biology, School of Biological Sciences, Ashworth Laboratories, University of Edinburgh, Edinburgh, Scotland; 2 Centre for Ecology and Hydrology, Bush Estate, Penicuik, Midlothian, Scotland

**Keywords:** Local adaptation, Ectomycorrhizal fungi (EMF), *Pinus sylvestris* (Scots pine), Heritability, Mutualism

## Abstract

Local adaptation of plants to mycorrhizal fungi helps determine the outcome of mycorrhizal interactions. However, there is comparatively little work exploring the potential for evolution in interactions with ectomycorrhizal fungi, and fewer studies have explored the heritability of mycorrhizal responsiveness, which is required for local adaptation to occur. We set up a reciprocal inoculation experiment using seedlings and soil from four populations of Scots pine (*Pinus sylvestris*) from Scotland, measuring seedling response to mycorrhizal inoculation after 4 months. We estimated heritability for the response traits and tested for genotype × environment interactions. While we found that ectomycorrhizal responsiveness was highly heritable, we found no evidence that pine populations were locally adapted to fungal communities. Instead, we found a complex suite of interactions between pine population and soil inoculum. Our results suggest that, while Scots pine has the potential to evolve in response to mycorrhizal fungi, evolution in Scotland has not resulted in local adaptation. Long generation times and potential for rapid shifts in fungal communities in response to environmental change may preclude the opportunity for such adaptation in this species, and selection for other factors such as resistance to fungal pathogens may explain the pattern of interactions found.

## Introduction

Mycorrhizal fungi are near-ubiquitous across the plant kingdom, forming associations with approximately 80% of plant species (Smith & Read, 2008). Despite our understanding of their importance in terrestrial ecosystems in terms of both host performance and soil nutrient cycling, it is only relatively recently that we are beginning to understand the evolutionary landscape that helps shape the outcomes of these interactions. In particular, local adaptation (the differential success of a genotype in its home environment compared with a foreign environment) has been explored as a potentially important process in structuring some of the context-dependency in host benefit seen in many mycorrhizal studies (Rúa et al., 2016b; Hoeksema, 2010). Clear evidence of the importance of these adaptive processes has been shown for plants forming arbuscular mycorrhizal (AM) symbioses (Rúa et al., 2016a; Johnson et al., 2010), but there have been comparatively few studies exploring local adaptation in ectomycorrhizal (EM) systems. While there is some evidence suggesting the existence of genotype × genotype interactions in ectomycorrhizal hosts (Piculell et al., 2008; Hoeksema & Thompson, 2007; Hoeksema et al., 2012; Pickles et al., 2015); overall, there appears to be little evidence so far for local adaptation in host populations (Rúa et al., 2018).

For local adaptation to occur, populations must possess sufficient heritable variation in fitness-related traits and experience differential selection pressures in different places (Cheplick, 2015). Theoretical work on coevolution in mycorrhizal interactions has usually considered factors affecting the outcomes of mutualism, such as partner selection, variation in symbiont community composition, and environmental quality as the drivers of selection (Hoeksema, 2010), resulting in pockets of local adaptation or maladaptation depending on the outcomes of these processes (the “geographic mosaic of coevolution”) (Thompson, 2005). While much work has used reciprocal transplants to investigate the potential for genotype × genotype interactions with mycorrhizal fungi (e.g. Hoeksema et al., 2012; Johnson et al., 2010), there have been comparatively few studies that have explored the heritability of the traits in question.

In particular, the heritability of ectomycorrhizal host traits, including host performance, degree of colonization, or compatibility traits, which are potentially important for local adaptation, has received only cursory attention. Many tree populations show very high amounts of genetic variation (Petit & Hampe, 2006; Cavers & Cottrell, 2015), which means that robust estimates of heritability will often require large amounts of replication both at the family and individual level, which can be time consuming to achieve. An even greater degree of replication may be required in situations where cloning of the host is difficult, such as in *Pinus* species. To our knowledge, only one study has estimated the heritability of host performance in response to inoculation, reporting high levels of heritability in *Pinus elliotii* inoculated with a single genotype EM inoculum (Rosado et al., 1994). Other studies have reported heritability in other mycorrhizal traits, such as the number of mycorrhizal root tips and degree of colonization (Rosado et al., 1994; Tagu et al., 2005; Courty et al., 2011; Velmala et al., 2012), and the heritability of ectomycorrhizal community composition on hosts (Lamit et al., 2016; Velmala et al., 2012).

Whether and how heritable variation in traits arises and is acted on by selection can also depend on factors other than those determining mutualistic outcomes. For example, life history strategies employed by the host or symbiont can change the landscape of adaptation through their effects on the amount and distribution of genetic variation (Leimu & Fischer, 2008; Cheplick, 2015). Mycorrhizal plants exhibit a wide range of life histories, from short-lived annuals to herbaceous perennials to long-lived trees (Smith & Read, 2008). The vast majority of ectomycorrhizal hosts are woody perennials, often trees (Smith & Read, 2008). As such life history factors, including long generation times, large numbers of offspring, and long distance gene flow, will have significant effects on the ability of such species to evolve in response to symbiotic partners (Petit & Hampe, 2006). It has been suggested that long generation times reduce adaptive potential, but phenotypic studies in trees often show strong differentiation between populations for adaptive traits, often with high values of heritability (Cavers & Cottrell, 2015). This differentiation likely results from strong selective pressures operating in the early stages of the life cycle when there is heavy mortality (Petit & Hampe, 2006; Cavers & Cottrell, 2015). For hosts to adapt in response to local EM fungal communities, there must thus be both heritable variation in the outcome of mycorrhizal interactions, and mismatched associations with local EM fungi must be deleterious to the fitness of seedlings. In addition, such deleterious effects should occur on similar scales to other selective pressures such as competition, grazing, and environmental stress.

Even in cases where populations show heritability for mycorrhizal traits, local adaptation still may only occur in situations where the identity and quality of their mycorrhizal associates are stable over the course of generations. If fungal community structure is liable to shift within the lifespan of a host, either through neutral processes or in response to shifting environmental conditions, then this can potentially disrupt any local adaptation that has occurred on the part of the host (Iason et al., 2018). Community turnover in EM fungi occurs not only in response to distance but also in response to environmental variation, including climatic variables such as annual rainfall and temperature, soil factors including pH, and in response to atmospheric deposition of N and K (Linde et al., 2018; Cox et al., 2010; Jarvis et al., 2013; Suz et al., 2014). EM fungi also show functional specialization on specific soil resources (Agerer, 2001). Community turnover is thus likely in response to shifting climatic conditions and increasing pollution (Cox et al., 2010; Suz et al., 2014). Under such changing conditions, local adaptation of host populations is likely impossible, and, instead, shifts in EM community composition in response to environmental change may help facilitate host survival if they are more able to provide host benefit under new conditions (Batstone et al., 2018; Iason et al., 2018).

To measure the heritability of host performance and test for local adaptation in an ectomycorrhizal host, we set up a reciprocal inoculation experiment using seedlings and soil from populations of Scots pine (*Pinus sylvestris*) from the Caledonian pinewoods of Scotland. Established approximately 10,000 years ago, these forests have experienced gradual historic climatic warming (Salmela et al., 2010) and currently occur in a wide variety of climatic conditions and altitudes across their limited range, with a west to east rainfall gradient of approximately 3000 mm to 500 mm per year over little more than 150 km (Donnelly et al., 2016). Local adaptation of these populations has previously been demonstrated along this climatic gradient (e.g. Salmela et al., 2011, 2013; Donnelly et al., 2016), and heritable variation has also been found for both resistance to needle pathogen *Dothistroma septosporum* (Perry et al., 2016a,b) and fungal endophyte community composition [Cavers, unpublished]. The forests also have a well-documented ectomycorrhizal flora, and EM community composition at these sites has been shown to vary strongly in response to both rainfall and altitude (Jarvis et al., 2013, 2015).

We sought to answer the following questions: (1) Is there heritable variation within Scots pine for response to inoculation by EM fungi? (2) Does this response differ depending on the pine population of origin? (3) If so, do populations perform better when paired with their local EM community compared to others?

## Methods

We set up a reciprocal cross-inoculation experiment to investigate the effects of soil biota on pine performance. The experiment used seedlings and soil from four populations of Scots pine across a longitudinal rainfall gradient in Scotland, resulting in a 4 × 4 factorial cross. To control for variability within populations and allow estimates of heritability, we used seed from six maternal families per population. Treatments consisted of seedlings grown in sterilized compost inoculated with field soil. Each treatment combination was replicated 8 times, with an additional 3 replicates containing sterilized inoculum to act as a control, resulting in a total of 1056 individuals. Plant performance was measured at the end of the experiment as aboveground and belowground biomass, and mycorrhizal colonization was measured for a subsample of root systems.

### Source material

**Fig. 1:**
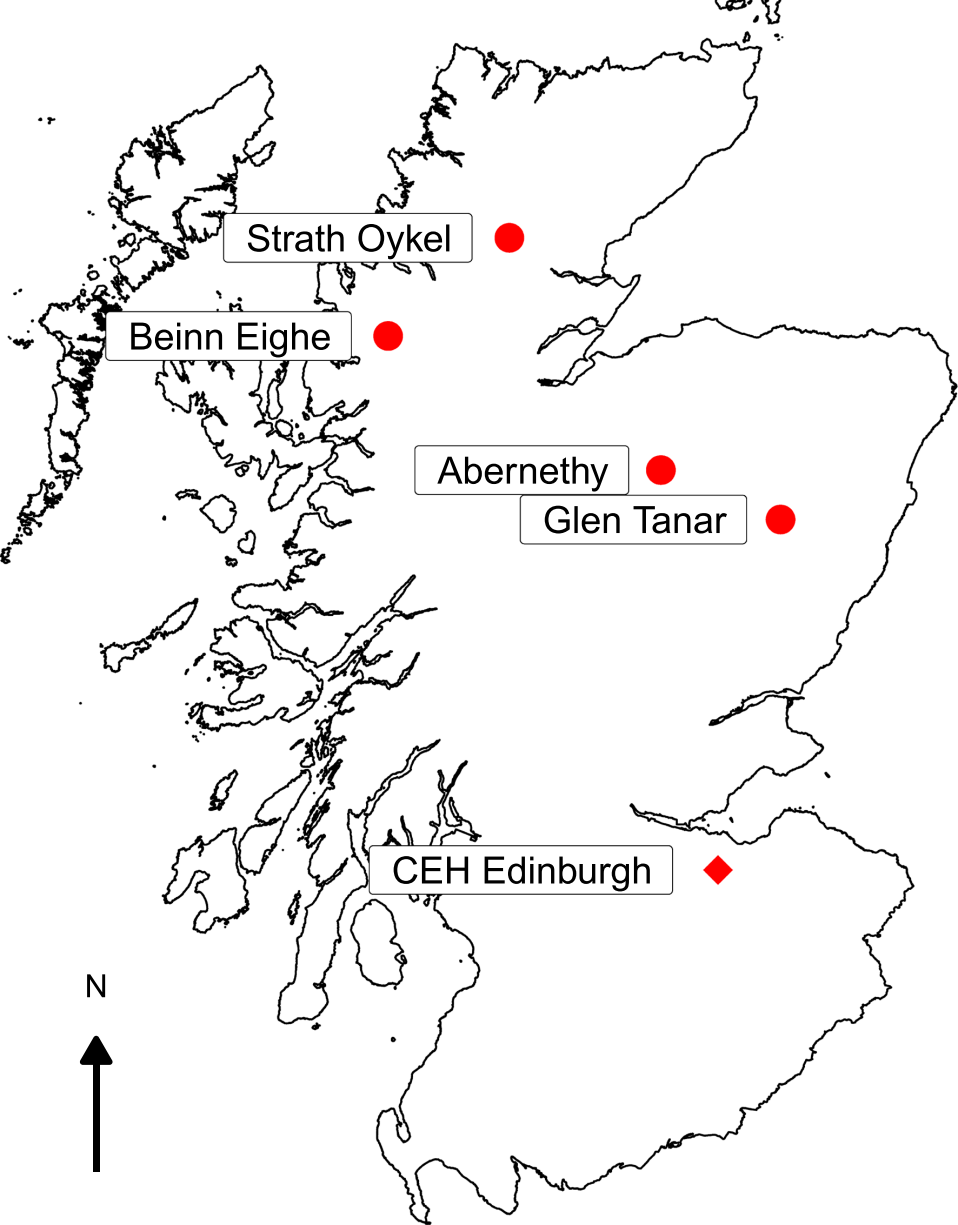
Map of each pine population used in the experiment (circles) and the location of the glasshouses (diamond) where the experiment was conducted

Cones were collected from four native stands of Scots pine in Scotland: Beinn Eighe and Strath Oykel in the west and Abernethy and Glen Tanar in the east (Fig. 1). These sites sit along a strong west-east rainfall gradient and additionally vary in both altitude and soil type (Table 1). Cones were collected from Beinn Eighe and Strath Oykel in late January 2018 and from Abernethy and Glen Tanar in late February of the same year. At each site, cones were collected from six open-pollinated mother trees. Seeds were cold-stratified at 4 °C for 3 weeks before being surface sterilized in 2% sodium hypochlorite for 10 min. They were then rinsed, imbibed in water for 3 h, and germinated on sterile agar before planting.

**Table 1:** Coordinates of each population used in the trial and the trial site, as well as climatic variables (Met Office) and soil types (Soil Information For Scottish Soils, James Hutton Institute). MAP = mean annual precipitation

Forest	Latitude	Longitude	Altitude (m)	MAP (mm)	Soil type
Beinn Eighe	57.63	− 5.35	21	2476	Peaty gleyed podzol
Strath Oykel	57.98	− 4.61	67	1234	Peaty gleyed podzol
Abernethy	57.21	− 3.61	362	1060	Humus-iron podzol
Glen Tanar	57.05	− 2.86	177	801	Humus-iron podzol
Common garden	55.86	− 3.21	190		

Field soil for use as inoculum was collected in late April 2018. At each site, soil from 4 small soil pits was collected to a depth of 30 cm from within the drip line of mature pine trees. These trees were chosen haphazardly, with a focus on collecting from beneath trees with low amounts of undergrowth, to allow for easy soil collection with minimal interaction with roots of other species. Collected soil for each site was sieved through a 6-mm mesh to remove stones and roots. The samples from each site were then homogenized to produce inocula for the experiment.

### Glasshouse set-up

Soil from each site was mixed with sterile potting soil (a compost:sand mix, 2:1 Levington stock nursery compost: Royal Horticultural Society Sharp Sand, sterilized by steam autoclave at 121 °C for 30 min) in a ratio of 7 potting soil:1 inoculum, and placed into 0.46-L square pots (9 × 9 × 9.5 cm). Germinated seedlings were cut out of agar and planted in the centre of the pot on the 31st May 2018. Each set of replicates was arranged randomly in blocks on two greenhouse benches, for a total of 8 blocks, 4 per bench; controls using sterilized inoculum (also autoclaved at 121 °C for 30 min) were placed into the blocks at each end of one glasshouse bench and at one end for the other. Pots were placed directly onto an unmatted flood bench base to limit root growth outside of the pots. After a week, any seedlings that had not taken hold were replaced with spares. Seedlings were watered in by hand following potting and subsequently were watered two to three times a day (depending on moisture levels) by an overhead sprinkler system. Seedlings were grown in this way for 4 months.

### Seedling analysis

Seedlings were harvested between the 18th of September and 1st of October 2019 in block order to capture any additional growth in the block effect. Seedlings were first cut at the soil level to separate above- and belowground biomass. Pots were then emptied, and the contents were carefully sieved and then washed to extract the roots with minimal damage. Root systems were inspected by eye for visible presence of mycorrhizal colonization—evident as thickened, coloured, and/or bifurcated root tips—and were scored for presence or absence. Subsequently, 5+ root systems per population:soil treatment were randomly selected from among 4 families per population for detailed analysis of root colonization. Colonization was assessed as the number of colonized ectomycorrhizal root tips per cm root length. Root length was measured following the gridline intersect method as described by Tennant (1975) using 5 randomizations. The shoots and roots for all seedlings were dried in an oven at 50 °C for 48 h, before being weighed to obtain a measure of above- and belowground biomass.

### Statistical analysis

In order to separate out the component of growth that was due to the soil biota, we used mycorrhizal response ratios instead of raw seedling measurements. These were calculated as the ratio of a seedling’s biomass or root:shoot ratio to the mean value of the sterile control seedlings for a given soil treatment and maternal family. In addition, we grouped soil treatments into two categories: western soils (Beinn Eighe and Strath Oykel) and eastern soils (Abernethy and Glen Tanar). This allowed us to simplify the interpretations of our model interactions to account for the major axis of variation between our sites. All statistical models were conducted in R using the *lmerTest* package.

#### Heritability and coefficient of genetic variation

We estimated narrow-sense heritability (*h*^2^), the proportion of phenotypic variance (*V*_*P*_) that can be explained by additive genetic effects, by fitting models for each trait in which maternal family and block were included as random effects Eq. 1, where *μ* is the mean and *ε* is the residual error. These models were fitted for each trait both within each inoculum treatment and for the full dataset.

1 $$ \mathrm{Trait}=\mu +\mathrm{Family}+\mathrm{Block}+\varepsilon $$

From these models, *h*^2^ was estimated using Eq. 2, where *V*_*A*_ is the additive genetic variance; *V*_*P*_ is the phenotypic variance; and *V*_fam_, *V*_block_, and *V*_res_ are the among family, among block, and residual variances, respectively; and *R* is the relatedness of individuals. As the relatedness of the seedlings was unknown, we estimated heritability for three values of *R*: seedlings are all half-siblings (*R* = 4), seedlings are 50% half-siblings and 50% full siblings (*R* = 3), and seedlings are all full siblings (*R* = 2).

2 $$ {h}^2=\frac{V_A}{V_P}=\frac{R{V}_{\mathrm{fam}}}{V_{\mathrm{fam}}+{V}_{\mathrm{block}}+{V}_{\mathrm{res}}} $$

Standard errors were calculated following Visscher (1998) (Eq. 3), where *s* is the mean number of offspring per family, and *f* is the number of families.

3 $$ S{E}_{h^2}=R\frac{\sqrt{2{\left(1-\frac{h^2}{4}\right)}^2{\left[1+\left(s-1\right)\frac{h^2}{4}\right]}^2}}{s\left(s-1\right)\left(f-1\right)} $$

We also calculated the coefficient of genetic variation (*CV*_*a*_) (Houle, 1992) (Eq. 4), a measure of genetic variation normalized by the trait mean. Heritability is an estimate only of the proportion of trait variance attributable to genetic components, so, by accounting for the total amount of phenotypic variance, *CV*_*A*_ provides a more realistic measure of the ability of a trait to respond to selection.

4 $$ C{V}_A=\frac{\sqrt{V_A}}{\mu_{\mathrm{Trait}}}\times 100 $$

#### Genotype × environment interactions

To investigate whether there were interactions between pine genotype and soil treatment, we used linear mixed modelling to test models structured as in Eq. 5. We included the specific soil origin as a random effect to control for variation between sites in our east/west soil categorization. In order to test for local adaptation, we used the same model structure but replaced the soil term with a term indicating sympatry or allopatry of the pine population and soil inoculum.

5 $$ \mathrm{Response}\sim \mathrm{Population}\times \mathrm{Soil}\left(\mathrm{east}/\mathrm{west}\right)+\mathrm{random}\left(\mathrm{Family}\right)+\mathrm{random}\left(\mathrm{Block}\right)+\mathrm{random}\left(\mathrm{Soil}\right) $$

## Results

**Fig. 2:**
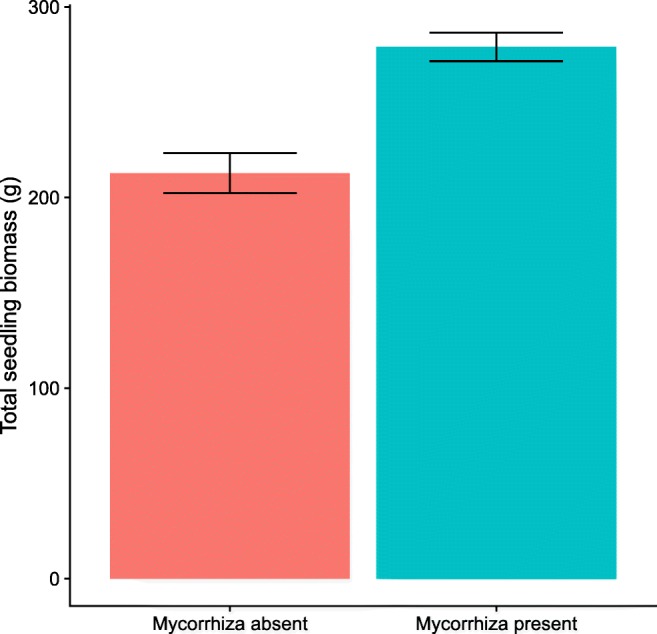
Bar chart showing the response of seedlings to the presence or absence of mycorrhizal fungi, regardless of mycorrhizal treatment. Error bars show 95% confidence intervals

In total, 1045 of 1056 seedlings survived through to the end of the experiment. Of these, 93% of inoculated seedlings showed signs of successful EM colonization, and 89% of sterile control seedlings remained uncolonized at the end of the experiment. Of the 81 seedlings for which we measured the degree of colonization, we found no effect of the degree of colonization on host biomass response (F_1,73_ = 1.2, *p* = 0.28). However, we did find that the presence of EM fungi on seedlings, regardless mycorrhizal treatment, had a net positive effect on growth (Mycorrhizal presence: F_1,1018.9_ = 25.9, *p* < 0.001; Mycorrhizal treatment: F_1,1013.5_ = 0.004, *p* = 0.95) (Fig. 2). For this reason, we assumed growth benefits to the seedlings were likely due to the presence of EM fungi rather than the other biotic components of the soil inocula. For all further analysis, we removed all seedlings from the data set that did not meet treatment standards. This left 960 seedlings in the dataset in total (709 inoculated seedlings, 251 control seedlings).

On average, seedlings inoculated with mycorrhizal fungi grew an additional 62.6 (SE ± 3.9) mg larger than non-inoculated seedlings. However, the range of responses was wide, with 29.3% of seedlings across all treatments showing reduced growth compared with the control. Additionally, inoculated seedlings allocated relatively less biomass to roots than shoots, with root:shoot ratios on average 7.5% (SE &plusmn 0.88%) smaller than those of control seedlings. Again, however, there was a wide range in the response of this trait, with 40.6% of all seedlings increasing their root:shoot ratio in response to inoculation.

### Heritability

**Fig. 3:**
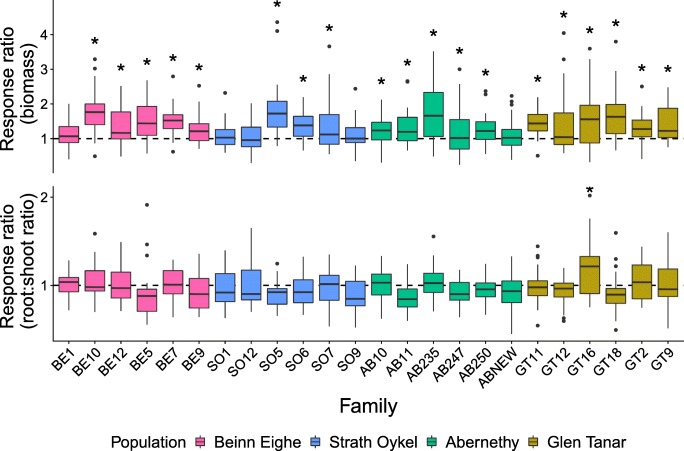
Box plots showing the range of response ratios of seedling families for biomass (top) and root:shoot ratio (bottom). Middle line shows the median, box shows the first and third quantiles, and the whiskers show no more than 1.5× the interquantile range. Colours indicate the population of origin for the seedling. Dashed line indicates no change compared with a non-inoculated seedling, with an asterisk indicating a significant difference compared with non-inoculated controls. Populations Beinn Eighe and Strath Oykel are located in the west, and Abernethy and Glen Tanar in the east

Although the amount of within- and between-family variation was very large (Fig. 3), we found evidence for significant heritability of both mycorrhizal response traits under all soil conditions (Table 2). Estimates varied from 0.13 to 1.05, although, in many cases, standard errors were large. Variation in traits due to block effects was low for all estimates; however, in all cases, residual variance accounted for the vast proportion of error.

**Table 2:** Heritabilities (SE) of mycorrhizal response traits in each of the three soil categories (western soils only, eastern soils only, and all soils). Each *R* column shows heritability estimated under different relatedness assumptions (*R* = 2: seedlings are full siblings; *R* = 3: seedlings are 50% full siblings and 50% half-siblings; *R* = 4: seedlings are all half-siblings). V columns: proportions of variance attributable to family (*V*_*f*_) and block (*V*_*b*_). *CV*_*A*_: Coefficient of genetic variation. We considered heritabilities to be significant when the standard errors did not overlap with zero

Trait	Soil origin	*h*^2^ (*R* = 2)	*h*^2^ (*R* = 3)	*h*^2^ (*R* = 4)	*V*_*f*_ (%)	*V*_*b*_ (%)	*CV*_*A*_
Biomass response	West	0.38 (0.09)	0.57 (0.16)	0.76 (0.24)	19.11	2.74	41.50
	East	0.52 (0.1)	0.78 (0.19)	1.05 (0.28)	26.17	2.89	41.36
	Both	0.2 (0.05)	0.3 (0.09)	0.4 (0.14)	10.06	2.51	28.24
Root:shoot response	West	0.32 (0.08)	0.47 (0.14)	0.63 (0.22)	15.79	3.31	17.89
	East	0.13 (0.06)	0.2 (0.1)	0.27 (0.15)	6.73	2.21	10.77
	Both	0.15 (0.04)	0.23 (0.07)	0.3 (0.12)	7.50	2.42	11.89

### Genotype × environment interactions

**Fig. 4:**
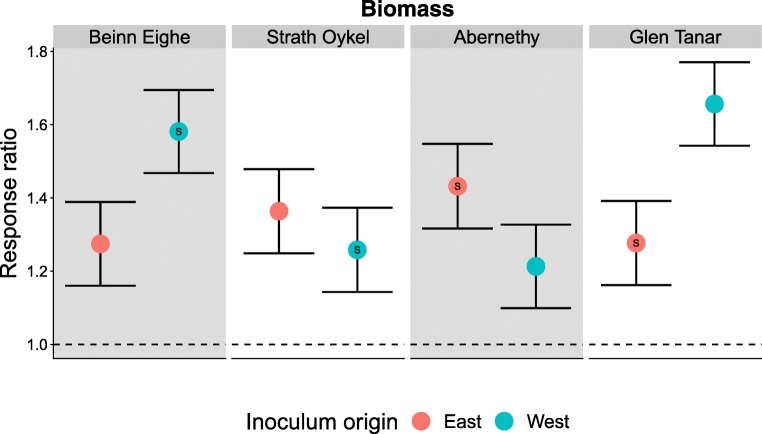
Response ratio of total seedling biomass ± SE from model predictions. Red points = eastern soil inoculum (Abernethy, Glen Tanar). Blue points = western soil inoculum (Beinn Eighe, Strath Oykel). S = sympatric combination. Pine populations are ordered from most western to most eastern. There were no significant differences apart from a significant interaction between Strath Oykel seedlings grown in western soil. Dashed line = no change compared with a non-inoculated seedling. Model predictions and SE were fitted using R package glmmTMB

We found evidence for a significant G × E interaction between seedling populations and soil treatments for biomass response to inoculation (Population × Soil: F_3,673.6_ = 12.6, *p* < 0.001), but no individual effects of population or soil (Fig. 4). In particular, we found that eastern inocula had a uniformly beneficial effect on growth for all populations, whereas western inocula had markedly different growth benefits depending on the population of origin for a seedling. Pines from either extreme of the longitudinal range in this study (Beinn Eighe in the west and Glen Tanar in the east) grew better with western inocula than eastern inocula, whereas pines from the middle two populations showed no difference in growth when inoculated with either inoculum.

**Fig. 5:**
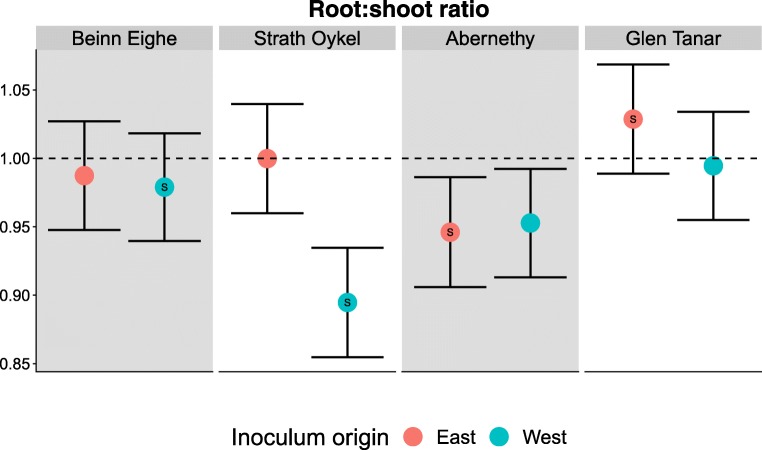
Percentage change in seedling root:shoot ratio following inoculation ± SE from model predictions. Red points = eastern soil inoculum (Abernethy, Glen Tanar). Blue points = western soil inoculum (Beinn Eighe, Strath Oykel). S = sympatric combination. The only significant interaction was a decrease in root:shoot ratio when Strath Oykel populations were grown in western soil. Pine populations are ordered from most western to most eastern. Dashed line = no change compared to a non-inoculated seedling. Model predictions and SE were fitted using R package glmmTMB

We also found evidence for a significant G × E interaction between population and soil treatment for a shift in root:shoot ratio following inoculation (Population × Soil: F_3,674.2_ = 2.7, *p* = 0.042), but no individual effect of soil or population (Fig. 5). More specifically, we found a complex pattern of interactions between soil types and populations: one population (Strath Oykel) allocated more biomass to shoots over roots when grown with western inocula, while the others (Abernethy, Beinn Eighe, and Glen Tanar) showed no differentiation in root:shoot response between inocula.

### Local adaptation

Assuming that the locally adapted phenotype would show increased biomass accumulation when grown with its local soil inoculum, we found no evidence for any main effect of growing in home soil vs away soil, either considering soils as four individual treatments (effect of sympatry on biomass response: F_1,674.8_ = 0.95, *p* = 0.33; effect of sympatry on root:shoot response: F_1,675_ = 3.07, *p* = 0.08) or on an east-west basis; effect of sympatry on biomass response: F_1,673.8_ = 0.06, *p* = 0.81; and effect of sympatry on root:shoot response: F_1,674.3_ = 2.1, *p* = 0.14).

## Discussion

Overall, we found evidence for heritability in both host performance and change in root shoot ratio in response to inoculation in all of our soil treatments. In line with previous studies in this system, we expected to find little overall differentiation between populations, but to find variation that fell along the major east-west climatic axis of Scotland. Although we did find evidence for G × E interactions between pine populations and soil inocula, suggesting some degree of genetic differentiation between populations in their response to specific fungi, we were unable to detect any clear pattern of geographic variation, either along the east-west gradient or in terms of local adaptation.

### Heritability

For both growth in response to inoculation and shift in root:shoot ratio, we found significant heritability within as well as across all soil treatments. Estimates of heritability were high under the most realistic relatedness scenario (*R* = 4, all half-sibs), but had wide estimated errors. Previously reported values for plant performance were broadly comparable with those found here for plant growth. Rosado et al. (1994) reported values of heritability for plant growth between 0.55 and 1.2 in inoculated seedlings, higher than in non-inoculated seedlings (0.26 to 1), in *Pinus elliottii* inoculated with *Pisolithus tinctorius*, suggesting a greater degree of variability in these traits in response to EM inoculation than without. However, the authors reported no errors for their estimates. A few other studies have shown heritability in other EM-associated host traits, including the number of EM root tips, root enzyme activity, and species-specific compatibility (Lamit et al., 2016; Tagu et al., 2005; Courty et al., 2011; Velmala et al., 2012), suggesting that host genotype is an important component of EM interactions. Understanding how these lower-level traits integrate to produce higher-level responses in host or fungal growth would be valuable to further our understanding of the process of adaptation in these organisms (Hoeksema, 2010).

Values of heritability are hard to accurately estimate and require large amounts of replication at the family and population level. Estimates can also be influenced by maternal effects; particularly, in the case of young seedlings, these effects can inflate measures of variance between families, leading to overestimation of heritability. Differences in environment between populations can also affect seed development, which may lead to additional differences in performance that are conflated with genetic variance (Falconer & Mackay, 1996). These latter effects are particularly difficult to control for without multigenerational trials, which are very time consuming when working with trees. We did not control for such non-genetic factors in this study, which may explain why our estimates of heritability were particularly high for our estimates of biomass response. It is also worth noting that the measurements of heritability in both this study and in Rosado et al. (1994) were conducted on seedlings grown in individual pots under glasshouse conditions. Seedlings in natural environments are normally connected to a common mycelial network, such that they are connected to fungal partners that are maintained by nearby mature trees (Teste et al., 2009). It has also been suggested that many of the negative outcomes seen in mycorrhizal studies are a result of such laboratory conditions and that experiments conducted in field conditions should produce more positive results (Frederickson, 2017). It would be instructive for future work to test for heritability of mycorrhizal response under field conditions as well.

### Local adaptation

In Scotland, mycorrhizal communities have been shown to vary strongly in response to the W-E rainfall gradient (Jarvis et al., 2013). Because of this, we assumed that community composition in the two western and two eastern inocula would be broadly similar. Our results here suggest that populations of Scots pine within Scotland have diverged in their interactions with EM fungi in terms of both growth and biomass allocation. This effect does not appear to be a simple effect of pine population, but instead depends on the specific combination of both population and inoculum in question. However, these G × E interactions show no clear geographic pattern. In particular, we can find no effect of either local adaptation or variation along the east-west climate gradient. Instead, the seemingly random pattern of interactions suggests that other unmeasured traits, such as resistance to fungal pathogens, may play a role determining the divergence of these populations.

Previous studies have reported mixed results for evidence of local adaptation in EM interactions. A previous study using 5 pine populations at a wider geographic scale found similar evidence for G × G interactions, but some host traits (relative growth rate and short root length) were smaller in sympatric plant-soil combinations (Hoeksema et al., 2012), while another study on the same system found a clinal gradient of colonization based on the distance between the host population and fungal population (Hoeksema & Thompson, 2007). A study explicitly incorporating climate, altitude, and soil effects also found that soil fungi mediated the adaptation of Douglas fir seedlings in over half of their treatment combinations (Pickles et al., 2015). However, a recent meta-analysis found no overall effect of host-fungal sympatry on host performance in EM systems, though this conclusion was based on a small number of studies (Rúa et al., 2018).

Because we sampled at a small spatial scale, we cannot exclude the possibility that gene flow swamped local selection. However, between the most eastern and most western populations, there is a lag of over 2 weeks in peak pollen production, which would limit gene flow between the geographic extremes (Whittet et al., 2017). Local adaptation has been demonstrated in these populations along the west-to-east gradient for a number of traits, including needle morphology (Donnelly et al., 2016), response to waterlogging (Donnelly et al., 2018), photochemical capacity (Salmela et al., 2011), and spring phenology (Salmela et al., 2013). Because mycorrhizal fungal communities in this pinewood system have also been shown to vary longitudinally along a rainfall gradient (Jarvis et al., 2013), we believe that the absence of local adaptation we found is due to a lack of selection for mycorrhizal response rather than the homogenizing effects of gene flow.

Instead, this overall lack of adaptation may result from life history traits of EM hosts. Over the course of a long lifespan, the importance of mycorrhizal associations for host fitness may vary strongly. For example, trees face varying strengths of intraspecific competition depending on their life stage (Petit & Hampe, 2006), and, thus, for a seedling, a boost in growth made by associating with preferred fungi may be much more important for survival than for a mature canopy tree. Alternatively, host preference in seedlings may be disadvantageous, as associations with whatever fungi are available may provide a better chance of survival than being selective. Additionally, if EM community composition is liable to shift within the lifespan of a host, then even if EM fungal associations pose a barrier to survival, then any host adaptation is likely to be quickly disrupted. Instead, the reassortment of EM community composition in response to environmental change may facilitate host survival by providing fungal species or genotypes that are better adapted to current conditions. EM fungi can manipulate host growth, for example by modifying the allocation of biomass to roots or shoots, which may facilitate host survival in a changing environment.

If local adaptation is unlikely in long-lived hosts, host-fungal genotype × genotype interactions may still be maintained through other traits that modify biotic interactions. Recent research is beginning to indicate that host resistance to pests and pathogens can influence mycorrhizal compatibility. For example, *Pinus edulis* individuals resistant to attack by a stem boring moth were found to harbour different EM communities (Sthultz et al., 2009), which provided differing amounts of host benefit under increasing drought (Patterson et al., 2018; Gehring et al., 2014). Similarly, genotypes of *P. taeda* resistant to two fungal pathogens showed both variation in compatibility with specific fungal genera and variation in relative growth rate (Piculell et al., 2018). Evidence of similar effects has also been hinted at in angiosperms, with fungal leaf pathogen communities strongly correlating with EM fungal communities (Lamit et al., 2015). Selection for resistance to these pathogens may thus help maintain the complex G × G interactions found in studies of local adaptation to EM fungi.

It is worth noting that the soil treatments applied here are likely to have included non-EM organisms, such as bacteria and pathogenic fungi. Although we found that the presence of mycorrhizal fungi generally resulted in an increase in plant growth, the G × E interactions found here could be influenced by negative interactions with soil-borne pathogens. If host pathogen resistance and EM response traits are indeed linked, then further work to separate these independent selective effects will be required. In the Caledonian pinewoods, resistance to the foliar needle pathogen *Dothistroma septosporum* has been found both among maternal families (Perry et al., 2016b) and among provenances (Perry et al., 2016a). Additionally, constitutive secondary defensive compounds, particularly monoterpenes, have been shown to influence herbivory by a variety of organisms (Iason et al., 2011), as well as the ground vegetation around a tree (Iason et al., 2005). Variation in the monoterpene *δ*^3^ − *carene* shows a geographic bias within Scotland, with high *δ*^3^ − *carene* levels uncommon in north-west sites. These or other genetic factors may play a role in determining response to mycorrhizal inoculation and could explain some of the G × E interactions found in this study. Further work investigating the effects of both *δ*^3^ − *carene* and monoterpene chemotype more broadly on Scots pine-EM interactions are underway at present.

## Conclusions

The complex set of host-soil interactions found here continues to underscore the complexity of geographic patterns in host-EM interactions. Although we were unable to ascribe a clear geographic pattern to the host-soil interactions found here, we demonstrated the heritability of mycorrhizal response in Scots pine within a relatively confined geographic area. Given the lack of data on the heritability of ectomycorrhizal responsiveness in the literature and the importance of heritability to the development of local adaptation, we would recommend that future studies on local adaptation to symbionts explicitly incorporate host genetic structure, such as clones or maternal families, in order generate robust estimates of heritability.
